# Periplogenin attenuates LPS-mediated inflammatory osteolysis through the suppression of osteoclastogenesis via reducing the NF-κB and MAPK signaling pathways

**DOI:** 10.1038/s41420-024-01856-0

**Published:** 2024-02-17

**Authors:** Kai Gan, Haoyu Lian, Tao Yang, Jian Huang, Junchun Chen, Yuangang Su, Jinmin Zhao, Jiake Xu, Qian Liu

**Affiliations:** 1 Guangxi Key Laboratory of Regenerative Medicine, Orthopaedic Department, The First Affiliated Hospital of Guangxi Medical University, Nanning, Guangxi 530021 China; 2 Collaborative Innovation Centre of Regenerative Medicine and Medical BioResource Development and Application Co-constructed by the Province and Ministry, Life Sciences Institute, Guangxi Medical University, Nanning, Guangxi 530021 China; 3 Faculty of Pharmaceutical Sciences, Shenzhen Institute of Advanced Technology, Chinese Academy of Sciences, Shenzhen, 518000 China

**Keywords:** Phenotypic screening, Mechanisms of disease

## Abstract

The key target for treating inflammatory osteolysis is osteoclasts. In an inflammatory environment, osteoclast differentiation increases, and bone resorption is enhanced. Periplogenin (Ppg) is a traditional Chinese medicine. It has anti-inflammatory and antitumor effects, but its impact on inflammatory osteolysis is unknown. This study found that Ppg prevented LPS-induced skull osteolysis by inhibiting the expression of inflammatory cytokines and osteoclast production. In vitro, Ppg blocked the RANKL-induced generation of osteoclasts, the development of pseudopodia bands, and bone resorption. Ppg also attenuated the expression of NFATc1, c-Fos, CTSK, and Atp6v0d2 proteins by inhibiting the NFATc1 signaling pathway. In addition, Ppg inhibited the expression of osteoclast-specific genes, including NFATc1, c-Fos, CTSK, Atp6v0d2, and Mmp9. Moreover, Ppg also inhibited NF-κB and MAPK pathways. In vivo, Ppg reduced the number of osteoclasts on the surface of the bone and suppressed LPS-induced osteolysis of the skull. These outcomes suggest that Ppg can serve as a new alternative therapy for treating inflammatory osteolysis by inhibiting inflammation and osteoclasts.

## Introduction

Maintaining the integrity of bone metabolic balance is coordinated by osteoclasts-mediated bone resorption and osteoblast-mediated bone formation [1]. Inflammation after tissue damage is essential for the early phases of repair, and its imbalance throws off the delicate process of bone remodeling [2]. Chronic inflammation suppresses osteoblast development, decreases bone production, and accelerates osteoclasts, causing increased bone resorption [3]. Variable inflammatory mediators play a crucial function in the deterioration of bone induced by osteoclasts [4]. When inflammation occurs near bones, some inflammatory osteolysis diseases arise. Examples include rheumatoid arthritis [5], psoriatic arthritis [6], periodontitis [7], periprosthetic osteolysis [8], and osteomyelitis [9], which can cause focal erosion, thus leading to severe consequences. Enhanced bone resorption and disruption of bone metabolic balance occur as a result of the activation and recruitment of osteoclasts, which is triggered by the production of inflammatory cytokines. This process is the primary cause of inflammatory osteolysis [10]. Potential mechanism-based treatment targets to stop inflammatory bone loss may be identified by gaining a deeper understanding of the mechanisms underlying bone resorption by osteoclasts and the cytokines that control their development and activity.

Osteoclasts are multinucleated large cells that arise from the fusion of monocyte/macrophage precursor cells produced from myeloid progenitor cells in the bone marrow. The growth and operation of osteoclasts are controlled by RANKL and macrophage colony stimulating factor (M-CSF) [11]. Stimulated by RANKL, the NF-κB and MAPK pathways are triggered to activate NFATc1 and c-Fos, an important component of the AP-1 transcription factor complex, to drive osteoclast differentiation [12]. In addition, signaling pathways such as ROS [13], PI3K-AKT [14], Notch [15], and Hippo [16] also promote osteoclast differentiation and activation. With the activation of signaling pathways, osteoclast-specific genes are also expressed, including, Ctsk, Atp6v0d2, and Mmp9 [17].

Although substantial medical advances have improved the treatment of inflammatory osteolytic disease, there are still certain drawbacks, such as the failure to repair broken bone and resistance to therapy in more than one-third of rheumatoid arthritis and psoriatic arthritis patients [18]. Recently, plant-derived natural products and their derivatives have proven valuable sources for exploring new avenues for treating clinical diseases due to their unique pharmacological activity [19]. Periplogenin (Ppg) is a natural furocoumarin in the root of Pycnogenol that has a highly effective anti-psoriasis effect [20] and anti-tumor [21] activity. Moreover, Ppg exhibits anti-inflammatory effects in psoriasis. Nevertheless, the impact of Ppg on inflammatory osteolysis triggered by osteoclasts is unknown. The current research proposed that Ppg may act as a novel therapeutic agent for inflammatory osteolysis by inhibiting osteoclastic formation. Therefore, the research aimed to investigate the possible effectiveness of Ppg in osteolytic bone conditions related to osteoclasts and to elucidate the potential molecular role of Ppg in osteoclastic formation.

## Results

### Ppg suppressed RANKL-induced osteoclast differentiation in vitro

**Fig. 1:**
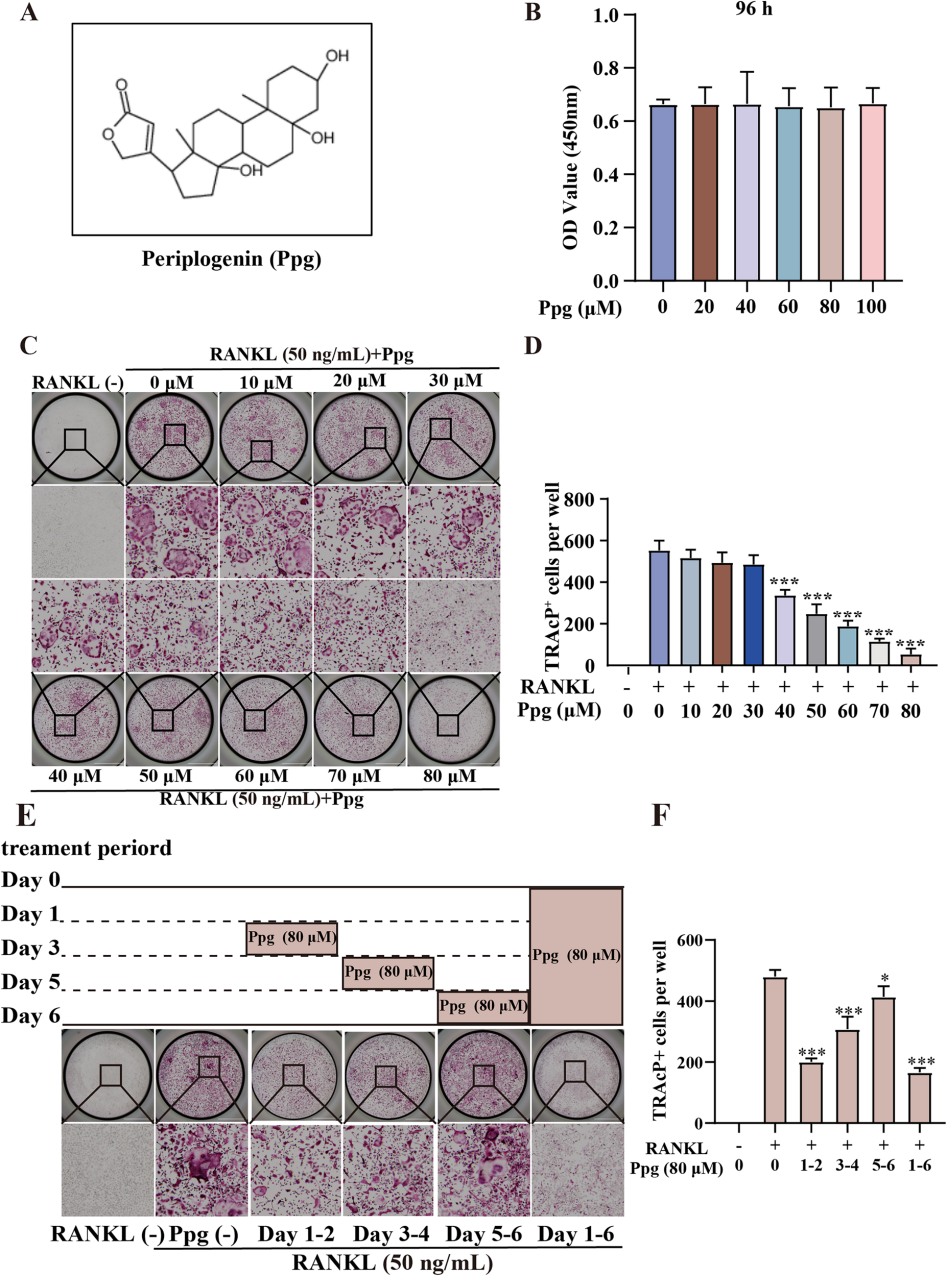
Ppg inhibited osteoclast formation induced by RANKL in vitro. **A** The chemical structure of Ppg. **B** After 96 h of treatment, the effect of different concentrations of Ppg on the cell viability of BMMs was determined by CCK-8. **C** BMMs were stimulated with RANKL for 5 days, and representative images of TRAcP staining showed that Ppg inhibited osteoclast formation in a dose-dependent manner. **D** Quantification of the number of TRAcP positive multinucleated cells (nuclei ≥3). **E** Effect of Ppg on osteoclast formation in a time-dependent manner. The representative image of TRAcP staining showed that Ppg (80 μM) was used to treat BMMs within a specified period of time during osteoclast formation (scale = 2 000 μM). **F** Quantification of the number of TRAcP-positive multinucleated cells (nuclei ≥3). The above data are expressed as the mean ± SD; *n* = 3; *p < 0.05, **p < 0.01 and ***p < 0.001. BMMs bone marrow macrophages, RANKL receptor activator of nuclear factor-κB ligand, TRAcP tartrate-resistant acid phosphatase.

Ppg has been found to exhibit anti-inflammatory and anti-tumor properties but its effect on osteoclasts is unclear. The current research assessed the impact of Ppg on osteoclast differentiation. First, the CCK-8 experiment revealed that Ppg did not inhibit bone marrow macrophages (BMMs) proliferation for 96 h at doses below 100 μM (Fig. 1B), indicating that Ppg was non-toxic to osteoclast survival. In addition, after a culture period of 5–7 days, it was observed that Ppg suppressed osteoclast differentiation in a dose-dependent manner (Fig. 1C, D). It was also observed that the inhibitory effect of Ppg was stronger during the early and middle phases of osteoclast development (Fig. 1E, F).

### Ppg suppressed osteoclast resorption function

**Fig. 2:**
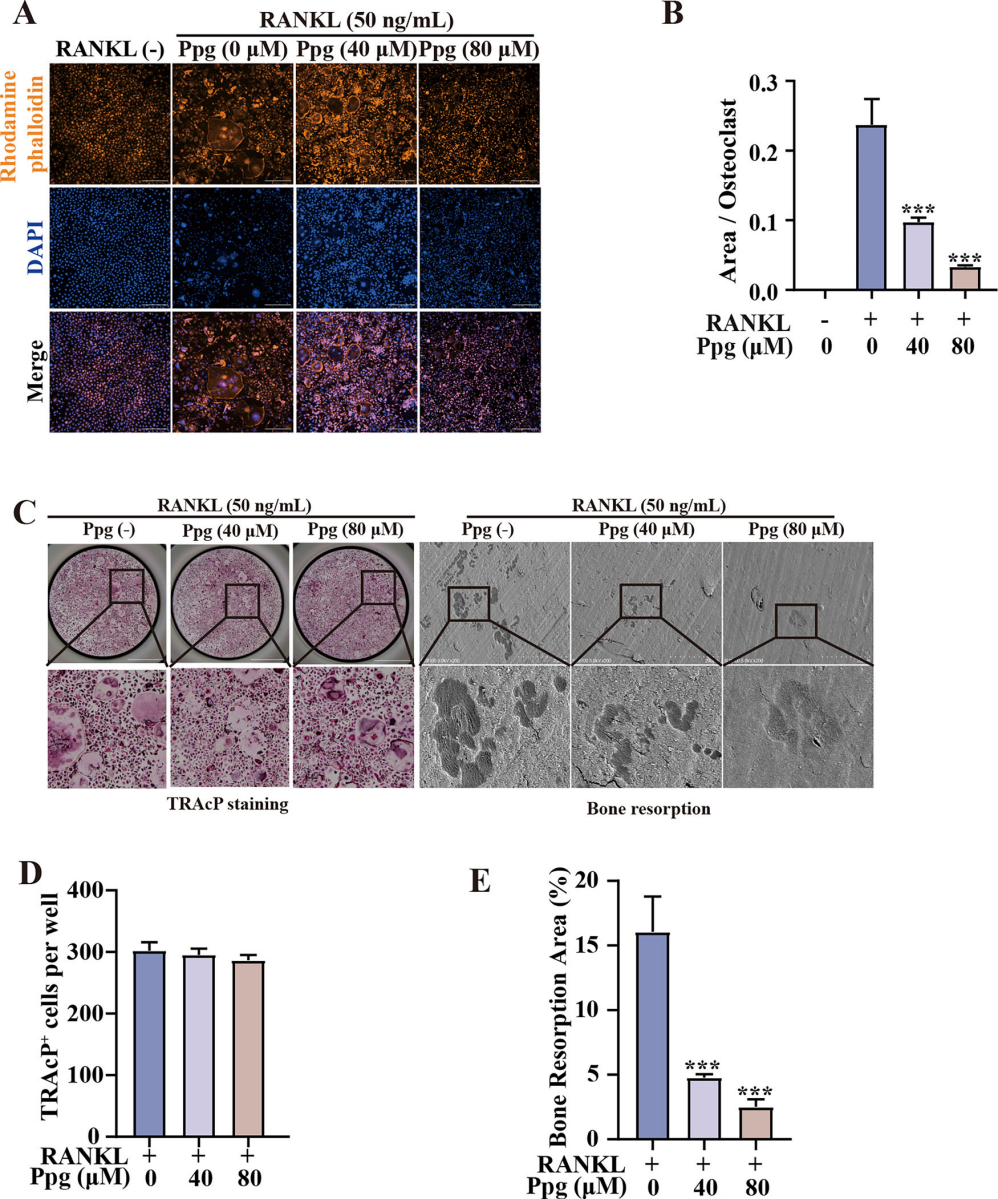
Ppg constrained osteoclast resorption function. **A** Immunofluorescence staining of f-actin ring and nucleus of mature osteoclasts. (The orange is the f-actin ring, and the blue is the nucleus, scale bar = 300 μm). **B** The relative quantity of osteoclast area in each visual field. **C** Representative images of TRAcP staining of Ppg-stimulated osteoclastogenesis (Scale bar = 2000 μm. The enlarged images Scale bar = 400 µm.) and electron microscope scanning of bone slice (Scale bar = 200 μm) in each group. **D** Quantification of the numbers of osteoclasts per well in each group (*n* = 3 per group). (**E**) Quantification of relative resorbed bone piece area per well in each group (*n* = 3 per group). All histograms are represented by mean ± variance. Compared with the RANKL induction group, *p < 0.05, **p < 0.01, ***p < 0.001.

Furthermore, Ppg-treated cells significantly reduced osteoclast size and osteoclast area compared to untreated cells (Fig. 2A, B). The bone resorption test showed that Ppg inhibited the resorptive function of osteoclasts on bovine bone sections (Fig. 2C–E). These results suggest that Ppg suppressed the resorptive function of osteoclasts.

### Ppg suppressed LPS-induced inflammatory response in BMMs

**Fig. 3:**
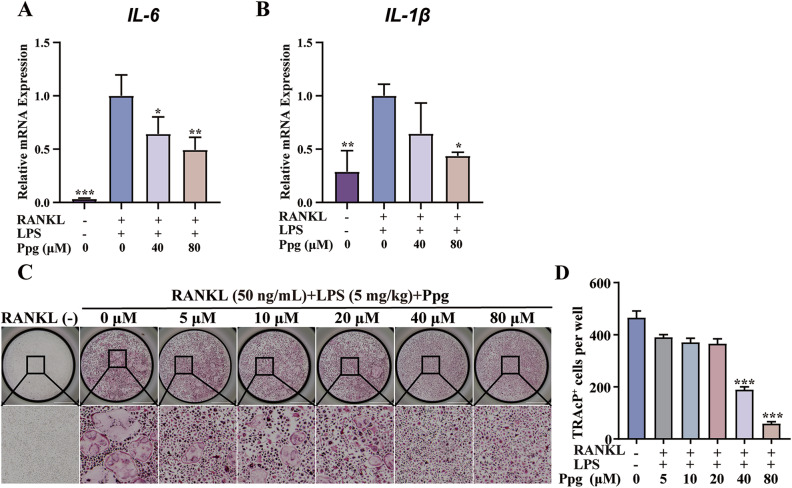
Ppg inhibited LPS-induced inflammatory response in BMMs. **A**, **B** Ppg inhibits the production of RANKL and LPS-mediated inflammatory factors IL-1β and IL-6. **C** Ppg inhibits RANKL and LPS-induced osteoclast production. **D** Quantitative analysis of the number of osteoclasts. The above data are expressed as the mean ± SD; *n* = 3; *p < 0.05, **p < 0.01 and ***p < 0.001.

Using a Real-time quantitative PCR assay, Ppg was found to inhibit the transcription of IL-1β and IL-6 in BMMs induced by LPS and RANKL (Fig. 3A, B). In addition, Ppg also attenuated LPS and RANKL-mediated BMMs differentiation of osteoclasts (Fig. 3C, D).

### Ppg suppressed LPS-induced osteolysis in mice in vivo

**Fig. 4:**
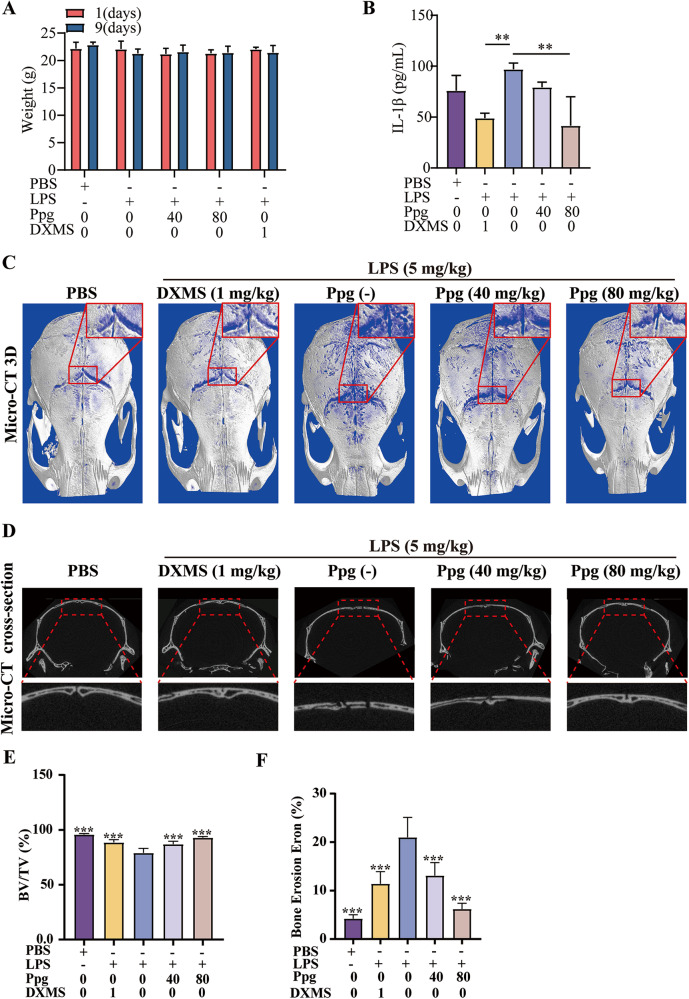
Ppg prevented LPS-induced osteolysis and bone loss in vivo. **A** Changes in mouse weight before and after modeling (*n* = 5). **B** Levels of IL-1β in LPS-induced calvarial osteolysis mice treated with or without Ppg were assessed by ELISA (*n* = 3). **C** Representative Micro-CT 3D constructional images showing that LPS-induced osteolysis was prevented by Ppg administration. **D** Representative cross-section images showing that LPS-induced osteolysis was by Ppg administration. **E**, **F** Quantification of BV/TV and osteolytic area in calvaria (*n* = 5). All of the above data are expressed as the mean ± SD, *n* = 5 per group. *p < 0.05, **p < 0.01 and ***p < 0.001. Relative to Vehicle group. BV/TV, bone volume/tissue volume values.

**Fig. 5:**
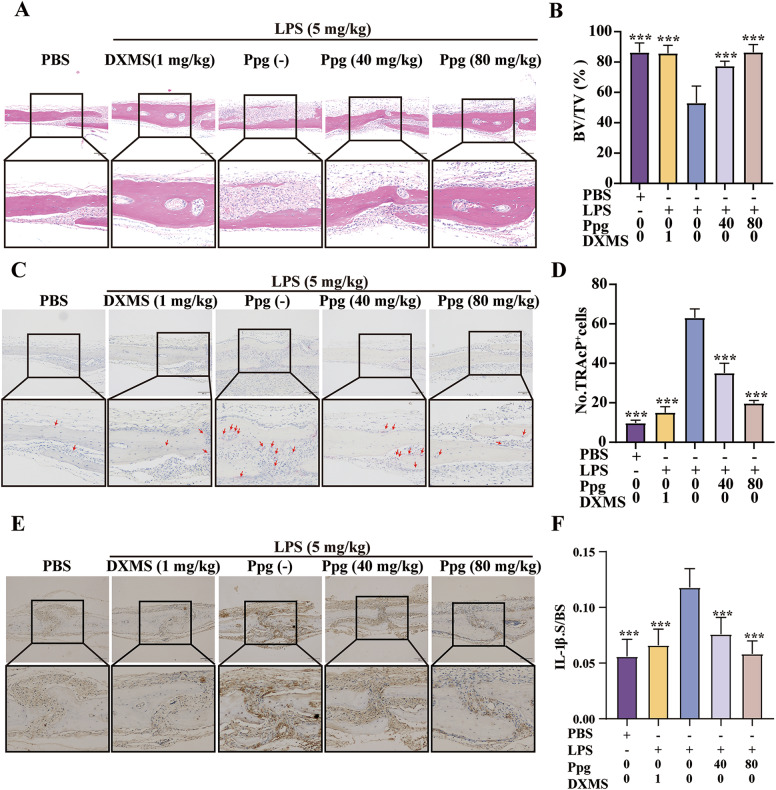
Ppg decreased osteoclast formation and production of pro-inflammatory cytokine in vivo. **A**, **C**, **E** Representative images of histomorphometry of mouse calvaria with H&E and TRAcP staining, as well as images of immunohistochemistry staining of IL-1β. **B** Quantification of BV/TV. **D** Quantitative analyses of numbers of TRAcP-positive cells. **F** Quantification of IL-1β-positive area (IL-1β.S). All of the above data are expressed as the mean ± SD, *n* = 5 per group; *p < 0.05, **p < 0.01 and ***p < 0.001. Relative to Vehicle group. H&E hematoxylin and eosin, IL-1β Interleukin-1β; TRAcP+, tartrate-resistant acid phosphatase.

To explore the impact of ppg on inflammatory osteolysis in vivo, an LPS-induced model of mouse skull osteolysis was used. The weight of the mice did not significantly alter over the course of the 9-day experiment (Fig. 4A), and no mice died or suffered any negative side effects. A previous report has highlighted that inhibiting the production of inflammatory environment and inflammatory factors can effectively relieve inflammatory osteolysis [22]. Based on the fact that DXMS can inhibit LPS-induced inflammation production, it was chosen as a positive control group in the current investigation [23]. IL-1β and IL-6 levels was assessed to determine the anti-inflammatory action of Ppg in vivo by using immunohistochemical labeling of mouse skull slices (Fig. 5E, F). Consistent with the findings obtained from in vitro experiments, the in vivo experiments also revealed that Ppg treatment lowered the levels of IL-1β protein expression (Fig. 4B). However, IL-6 levels did not change (Supplementary Fig. 1). Micro-CT scanning of the isolated mouse skulls showed that the skull osteolysis of mice after Ppg treatment was significantly reduced (Fig. 4C–F). Consistently, histology analyses showed that the BV/TV volume of mice in the high-dose Ppg group was comparable to the sham groups (Fig. 5A, B). Ppg dramatically decreased the TRACP-positive cell/bone surface ratio and the overall number of osteoclasts per field in comparison to the LPS group (Fig. 5C, D). The overall findings of the current research imply that Ppg inhibits osteoclast formation, has anti-inflammatory effects in vivo, and might inhibit LPS-mediated inflammatory bone loss.

### Ppg downregulated the expression of genes and proteins specific to osteoclasts during osteoclastic development

**Fig. 6:**
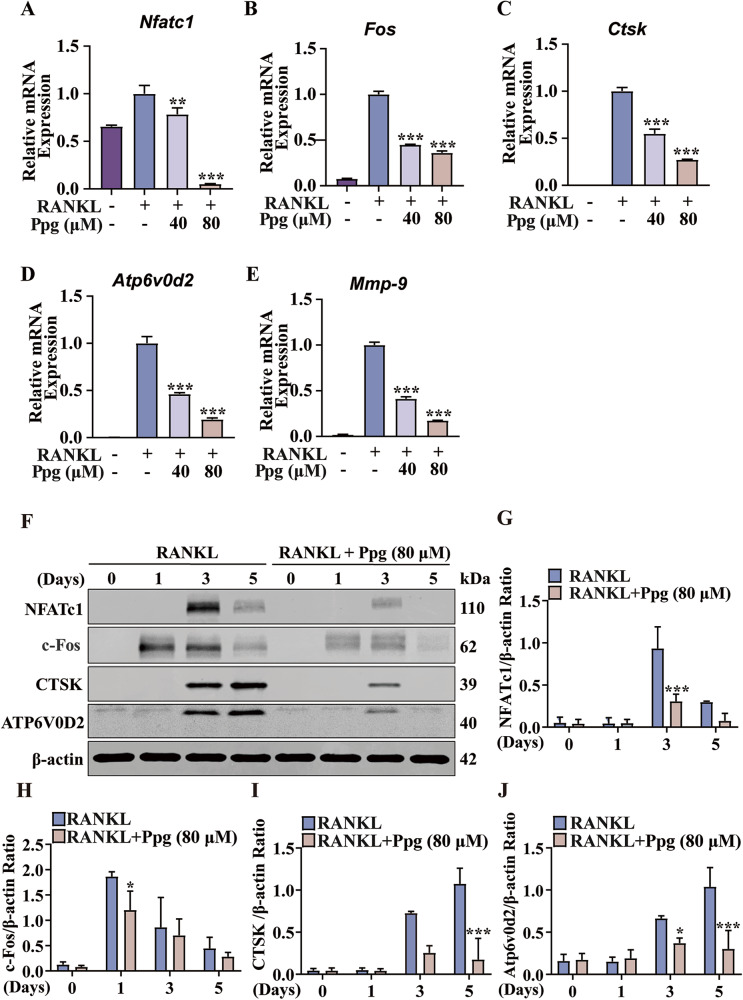
Ppg downregulated osteoclast-specific genes and proteins during osteoclastogenesis. **A**–**E** The expression of Nfatc1, c-Fos, Ctsk, Atp6v0d2 and Mmp9 were assessed by RT-qPCR, β-actin serves as a loading control (*n* = 3). **F**–**J** The expression of NFATc1, c-Fos, CTSK and Atp6v0d2 proteins were assessed by Western blot. All bar graphs are shown as mean ± SD. *p < 0.05, **p < 0.01 and ***p < 0.001 compared with the control group (treated with RANKL but without Ppg). BMMs bone marrow macrophages, Nfatc1 nuclear factor of activated T cells 1, RANKL receptor activator of the nuclear factor-κB ligand, c-Fos Proto-oncogene C-Fos, Mmp9 matrix metallopeptidase 9, Ctsk cathepsin K.

Ppg was found to inhibit the expression of genes unique to osteoclasts, including c-Fos, NFATc1, CTSK, Atp6v0d2, and MMP9 by Real-time PCR (Fig. 6A–E). Consistently, it was observed that Ppg also inhibited the protein expression of c-Fos, NFATc1, Atp6v0d2, and CTSK during osteoclast differentiation (Fig. 6F–J). The outcomes highlight that Ppg suppressed the expression of genes and proteins specific to osteoclasts, thereby suppressing the differentiation and functioning of osteoclasts.

### Ppg inhibited RANKL-induced NF-κB and MAPK pathways in osteoclast differentiation

**Fig. 7:**
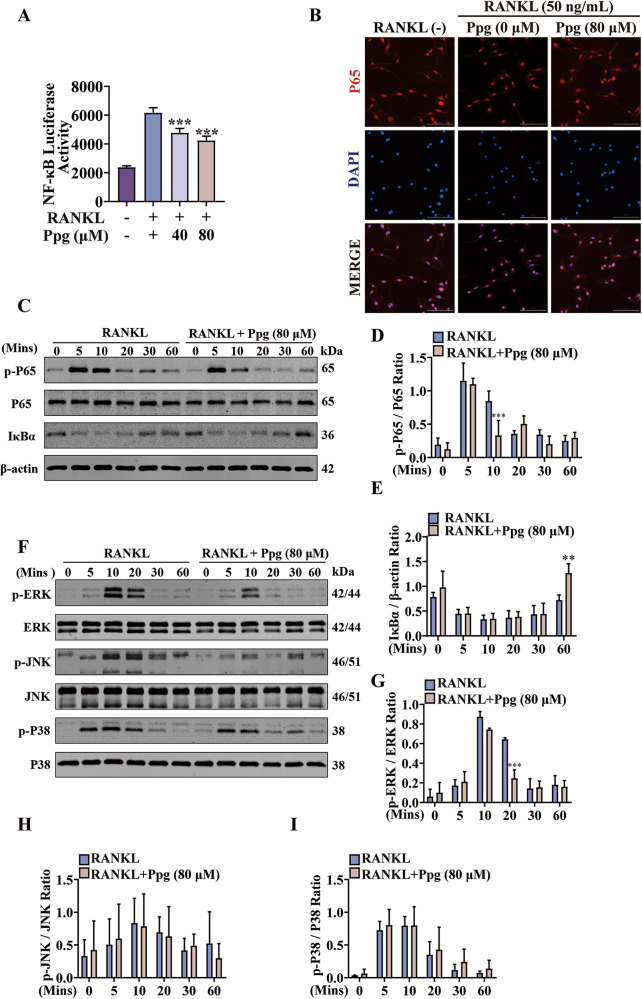
Ppg interfered with RANKL-induced activation of NF-κB and MAPK-ERK signaling pathways. **A** Immunofluorescence images of p65 nuclear translocation following RANKL stimulation without or with 80 μM Ppg treatment (scale = 100 μM). Cell nuclei were counterstained with DAPI. **B** NF-κB luciferase assay showing that Ppg inhibited NF-κB transcriptional activity dose-dependently (*n* = 3 per group). **C**, **F** The expressions of NF-κB and MAPK proteins induced by RANKL in 0–60 mins were assessed by Western blot. **D**, **E** The ratios of p-P65/P65 and IκBα/β-actin (*n* = 3). **G**–**I** The ratios of p-ERK/ERK, p-JNK/JNK, p-P38/P38 (*n* = 3). Bar graphs are shown as mean ± SD. *p < 0.05, **p < 0.01, ***p < 0.001 compared with the control group (treated without Ppg).

**Fig. 8:**
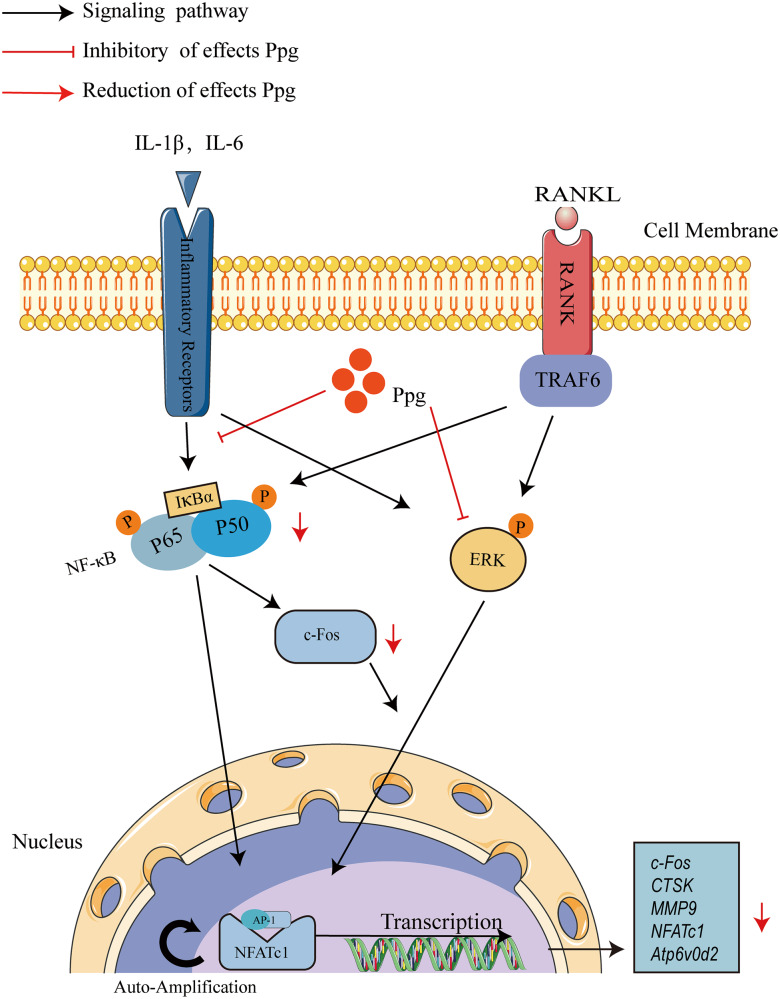
A proposed working model for the inhibitory effects of Ppg on osteoclastogenesis. Ppg Periplogenin, NFATc1 nuclear factor of activated T cells 1, c-Fos Proto-oncogene C-Fos, Ctsk cathepsin K, Mmp9 matrix metallopeptidase 9, RANKL receptor activator of nuclear factor-κB (NF-κB) ligand, NF-κB nuclear factor-κB, MAPKs mitogen-activated protein kinases, Atp6v0d2 ATPase H+ transporting V0 subunit d2.

Next, the effect of Ppg on NF-κB and MAPK pathways during osteoclast differentiation was investigated. The results of the luciferase reporter assay showed that treatment with Ppg inhibited the activation of NF-κB (Fig. 7A), and at the same time, Ppg significantly blocked P65 nuclear translocation (Fig. 7B). Consistent, western blot analysis showed that p-P65 (Fig. 7C, D) and degradation of IκBα were also significantly inhibited (Fig. 7C, E). Similarly, p-ERK was substantially suppressed 20 min after stimulation of RANKL (Fig. 7F, G). In comparison, Ppg had no significant effect on p-P38 and p-JNK on BMMs mediated by RANKL at 0–60 min (Fig. 7F, H, I). These data suggest that Ppg suppressed osteoclast production by regulating NF-kB and MAPK pathways. Next, we detected p-AKT protein expression and measured intracellular ROS levels. The results showed that the expression of p-AKT protein was not inhibited, and the ROS level was not significantly changed (Supplementary Fig. 2). Figure 8 presents the schematic mechanism of the inhibitory effect of Ppg on osteoclastogenesis.

## Discussion

The hunt for novel medications to limit osteoclast function is a research hotspot for osteoclast-related osteolytic disorders since osteoclasts are essential cells implicated in bone loss. As a result of inhibiting the pro-inflammatory cytokines IL-1 and IL-6 and deactivating the NF-κB and ERK pathways, Ppg was able to suppress osteoclast differentiation and function in vitro. Further, the ability of Ppg to dampen LPS-induced inflammatory osteolysis was also highlighted.

Ppg was isolated from the root of a traditional Chinese medicinal plant called Periploca sepium Bunge and is recognized as one of the key components of Cortex Periplocae [24]. The root bark of Periploca sepium Bunge, often called Cortex Periploca (Xiangjiapi in Chinese), has been commonly utilized for treating rheumatoid arthritis and promoting bone strength [20]. Ppg also safeguards against hyperthyroidism and associated cardiovascular illness, according to several pharmacological trials [25]. In addition, there have been reports of anti-inflammatory activity and anticancer properties of Ppg in recent years [26]. The current study revealed its novel effect on osteoclast differentiation and function via dampening RANKL-induced osteoclast production, podosome belt formation, and bone resorption.

The critical pathways such as MAPK and NF-κB, activated by RANKL during osteoclastogenesis, have been extensively studied [27, 28]. These pathways were key downstream cascades of TRAF6 activated by RANKL/RANK axis [29]. NF-κB is a significant factor in osteoclast development and is involved in osteolytic bone diseases [30, 31]. Phosphorylation of IκBα by the classic pathway and its degradation by the proteasome during osteoclast differentiation result in the translocation of the NF-κB dimer (p50 and p65/relA) into the nucleus [32]. Extracellular signal-regulatory kinases 1 and 2 (ERK1/2) in the MAPK pathway are triggered by the sequential activation of MKKK, MKK, and MAPK [33]. Activation of ERK, especially ERK1, can promote phosphorylation of transcription factors NFATc1 and c-Fos, thereby increasing their transcriptional activity and promoting osteoclast differentiation [34]. Relevant studies have confirmed that the PI3K/AKT signaling pathway is involved in osteoporosis, osteoarthritis, osteosarcoma, and other pathological bone diseases by regulating the proliferation, differentiation, and apoptosis of osteoclasts and osteoblasts [35]. In addition, ROS is a multifunctional signaling molecule in the intercellular pathway during osteoclast differentiation [36].

Our results suggest that Ppg regulates osteoclast differentiation and function through the NF-κB and MAPK signaling pathways rather than the PI3K-AKT and ROS signaling pathways. It is worth noting that Ppg is also a STAT3 inhibitor that directly and specifically binds to STAT3, resulting in the downregulation of STAT3 phosphorylation at Tyr705, thereby reducing downstream cascade dimerization, translocation, and function [21]. Activation of STAT3 has been shown to induce RANKL production and stimulate osteoclast formation [37]. Further investigation should focus on the interaction between Ppg and STAT3 in osteoclasts.

Furthermore, NFATc1 is an indispensable transcription factor for regulating osteoclast differentiation [38]. The cytoplasmic version of NFATc1 is persistently present and strongly phosphorylated. When it is triggered by RANKL or its downstream signaling pathway, its serine residues are dephosphorylated and moved into the nucleus [39, 40]. Following its entry into the nucleus, NFATc1 collaborates with other transcription factors to increase the expression of genes unique to osteoclasts, including Ctsk, Atp6v0d2, and Mmp9, thereby boosting osteoclast differentiation and maturation [41]. One of the key roles of c-FOS in osteoclast formation is to produce NFATc1, which can work together to start a transcriptional regulatory cascade that activates several target genes associated with osteoclast development and function [42]. This research demonstrates that Ppg can dampen NFATc1 and c-Fos mRNA and protein expression, contributing to the inhibition of osteoclast formation and function.

Inflammation activates ERK and NF-κB signaling pathways during osteoclastogenesis [43]. Bacterial endotoxins, including LPS, are thought to be the primary mediators of inflammatory osteolysis [44]. LPS induces the production of osteoclast cytokines, including MCSF and the NF-κB ligand RANKL, which directly stimulate osteoclast formation and activity, resulting in catastrophic bone loss [45, 46]. Moreover, LPS is a powerful inducer of inflammation and pro-inflammatory cytokines that stimulate macrophages, fibroblasts, and other cells for secreting multiple pathogenic cytokines, including IL-1β and IL-6 [47, 48]. Therefore, this study verified that Ppg can inhibit the inflammatory factors IL-1β and IL-6 in vitro. Ppg inhibits IL-1β in vivo but has no effect on IL-6, which might be because the environment in vivo is coordinated by a variety of factors, and cultured cells in vitro cannot fully simulate the state in vivo. The anti-osteolytic action of Ppg was then confirmed using a mouse model of LPS-induced skull osteolysis to imitate clinical inflammatory osteolysis. In line with our results, previous studies have found that Ppg is able to improve psoriasis-like skin inflammation induced by IMQ [20].

The inflammatory osteolysis process involves not only the activity of osteoclasts but also the effect of delayed differentiation of bone marrow mesenchymal cells [49]. We also assessed the impact of Ppg on osteoblastogenesis through ALP analysis. Our findings indicate that Ppg does not exert a significant influence on osteoblast differentiation (Supplementary Fig. 3).

In conclusion, these findings offer encouraging evidence that Ppg is capable of inhibiting inflammation-mediated osteoclastogenesis and thus holds as a therapeutic candidate for treating inflammatory osteolytic illness associated with the overactivation of osteoclasts.

## Materials and methods

### Media and reagents

Ppg (HPLC98%) was provided by the Chengdu Must Biological Technology Co., Ltd. (China) for this study. Fetal bovine serum (FBS), penicillin-streptomycin (PS) solution, and alpha-modified minimum essential medium (α-MEM) were supplied by Gibco (Thermo Fisher Scientific, United States). Dimethyl sulfoxide (DMSO) was used to dissolve Ppg to a storage concentration of 100 mM before diluting Ppg to different concentrations by adding α-MEM. Moreover, Cell Counting Kit-8 (CCK-8) was supplied by MedChemExpress (MCE, China). Recombinant M-CSF and RANKL mice were supplied by R&D Systems (USA). Specific primary antibodies, such as p-P65, P65, phosphorylated (p)-ERK, ERK, p-JNK (c-Jun N-terminal kinase), JNK, p-P38, P38, β-actin, V-ATPase d2, and c-Fos, as well as secondary antibodies, such as rabbit and mouse, were supplied by Cell Signaling Technology (USA). Furthermore, Abcam (UK) provided the antibodies against CTSK, IκBα, and NFATc1.

### Cell culture and osteoclast differentiation assay

The Animal Center of Guangxi Medical University (China) provided male C57BL/6J mice 6–8 weeks old. Primary bone marrow macrophages (BMMs) were obtained from the femur and tibia of mice via bone marrow lavage. Culturing of BMMs was done in a complete medium containing 10% FBS, 1% PS, and 25 ng/mL M-CSF in α-MEM at 5% CO_2_, a temperature of 37 °C, and placed in a T75 flask (USA). Following a 48-h incubation, 7 × 103 cells/well were inoculated into a 96-well plate. On the second day following cell attachment, osteoclast differentiation was induced by adding an osteoclast culture medium containing RANKL and M-CSF, as per a previous report [50]. Subsequently, varying concentrations of Ppg (10, 20, 30, 40, 50, 60, 70, or 80 μM) were added within six days. BMMs were treated with Ppg (80 μM) at several stages of osteoclastogenesis for investigating the impact of Ppg on the differentiation of osteoclasts. BMMs were rinsed with PBS and fixed with 4% PFA, followed by staining with TRAcP reagents (Sigma, USA). Mature osteoclasts refer to fusion cells with a minimum of three nuclei. Images of the samples were captured using a Bio-tek microscope (VT, USA), and the osteoclasts were quantified by utilizing Image J (NIH, Bethesda, USA).

### Cell viability/cytotoxicity assay

Cytotoxicity of Ppg was detected with the aid of the CCK-8 kit. BMMs (7 × 103 cells/well) were seeded into 96-well plates. The following day, cells were stimulated with varying concentrations of 20, 40, 60, 80, or 100 μM for 96 h. Next, 10 μL of CCK-8 reagent was introduced into each well before incubating under 37 °C and 5% carbon dioxide for 2 h. The OD value of each well was computed at 450 nm by employing a TriStar2 LB 942 Microplate Reader (Berthold Technologies Gmbh&Co.KG, Germany).

### Staining test for podosome belt formation

Culturing and treatment of BMMs were done with Ppg (0, 40, and 80) in 96-well plates. As previously described [51], staining, detection, and imaging of mature osteoclasts were done using RGB and DAPI channels with the aid of a Biotek microscope.

### Bone pits resorption assay

In the control group, the labeled beef bone slices were placed in 96-well plates. The BMMs were seeded into each well at a density of 8 × 103 cells per well. BMMs were then stimulated with RANKL (50 ng/mL) until microscopic osteoclasts started to appear within the wells. Subsequently, the individual osteoclasts formed in each well were treated with Ppg (0, 40, and 80 μM) for a duration of 48 h. Following this treatment, bovine bone slices were fixed using a solution containing 2.5% glutaraldehyde from Solarbio (China), and their absorption area was determined by scanning electron microscopy (SEM). Furthermore, 4% PFA cells were fixed in control wells for 10 min, followed by staining using TRAcP to analyze osteoclast differentiation. Quantification of bone resorption pit regions and TRAcP-positive cells was performed using image J.

### LPS-induced calvarial osteolysis mice model

The Laboratory Animal Ethics Committee of Guangxi Medical University granted its approval for all in vivo experiments. The protocol for appropriate care and use of laboratory animals was followed while carrying out these experiments. Sample sizes for animal studies are determined based on an examination of data from published studies or preliminary investigations. Twenty-five male C57/BL6 mice aged 8 weeks were classified into five groups: sham (PBS injection daily), dexamethasone (DXMS) (alternating LPS 5 mg/kg and DXMS daily), LPS (lipopolysaccharide (LPS) 5 mg/kg alternating and PBS alternately), and Ppg (alternating LPS 5 mg/kg and Ppg 40 and 80 mg/kg). For eight consecutive days, 100 μL of relevant reagent was injected subcutaneously every day around the V-suture of the skull. On day 9, mice were euthanized by cervical dislocation. Subsequently, the skulls were isolated and immobilized using 4% formalin for further analysis. Prior to receiving any treatment, each animal was randomly assigned to a group. Mice were treated in a blinded fashion as the drugs used for treating animals were prepared by researchers who did not carry out the treatments.

### Micro-CT scanning

Mice skulls immobilized with 4% PFA were scanned at 50 kV and 500 μA using the Skyscan 1176 micro-CT system (Bruker, USA), with additional parameters including a 9 μm pixel size, a 0.5 mm AI filter, and a 180-degree rotation step. The SkyScan NRecon platform was utilized to reconstruct three-dimensional (3D) images, which were then analyzed using SkyScan CTAn software (Bruker).

### Histological examination

The mouse skull was subjected to micro-CT scanning and subsequently placed in 14% EDTA for a duration of 21 days. Furthermore, the mouse skull was embedded in paraffin and cut into 5-micron sections before being exposed to various staining techniques including TRAcP activity staining, hematoxylin-eosin (HE) staining, and immunohistochemical staining for further observation and analysis.

### Real-time quantitative PCR assay

BMMs were seeded into a six-well plate at a density of 1.5 × 105 cells/well, and cells were induced with different Ppg (0, 40, 80 μM) until osteoclasts appeared. The total RNA extraction for individual samples was done by utilizing Trizol. Reverse transcription was conducted to generate cDNA as per the provided instructions, as a template for Real-time quantitative PCR. The following cycle parameters were set on the LightCycler ®96 system (Roche, Switzerland): 94 °C for 10 min, followed by 45 cycles at 95 °C × 15 s and 60 °C × 60 s. All data were normalized to β-actin using comparative threshold cycling. The primers utilized in the current investigation are presented in Table 1.

**Table 1:** The primers used in this study.

Target Gene	Primer Sequence (5ʹ- 3ʹ)	
	Forward	Reverse
Fos	CCAGTCAAGAGCATCAGCAA	AAGTAGTGCAGCCCGGAGTA
Nfatc1	GGTGCTGTCTGGCCATAACT	GAAACGCTGGTACTGGCTTC
Ctsk	AGGCGGCTATATGACCACTG	TCTTCAGGGCTTTCTCGTTC
Mmp9	GAAGGCAAACCCTGTGTGTGTT	AGAGTACTGCTTGCCCAGGA
Atp6v0d2	GTCCCATTCTTGAGTTTGAGG	GGATAGAGTTTGCCGAAGGTT
β-actin	TCCTCCCTGGAGAAGAGCTA	ATCTCCTTCTGCATCCTGTC
Il1β	GCAACTGTTCCTGAACTCAACT	ATCTTTTGGGGTCCGTCAACT
Il6	TAGTCCTTCCTACCCCAATTTCC	TTGGTCCTTAGCCACTCCTTC

### Western blot

BMMs in each well were lysed on ice with radioimmunoprecipitation assay (RIPA) lysis buffer containing 1% phenylmethylsulfonyl fluoride (PMSF), 1% phosphatase, and 1% protease inhibitor for a minimum of 30 minutes. After collecting the lysate, it was subjected to centrifugation at a rate of 12,000 rpm for 10 min. The extracted protein was then quantified by utilizing the BCA Protein Assay Kit (Beyotime, China). After mixing the prepared protein sample with sodium dodecyl sulfate (SDS) loading buffer, it was heated at 98 °C for 20 min. Moreover, the protein samples were transferred to a nitrocellulose membrane through an electrical transfer process after separating them by 10% SDS-polyacrylamide gel electrophoresis (SDS-PAGE). Following the blocking of non-specific binding immune response using 5% skim milk in 1×TBST, the nitrocellulose membrane was soaked in a primary antibody and kept in an incubator at 4 °C with gentle shaking for a period of 12–14 h. Subsequently, the nitrocellulose membranes were washed thrice with 1×TBST, followed by soaking in the corresponding secondary antibody, and kept at room temperature for 1 h. Lastly, images of protein bands were captured by employing the Odyssey® DLx Imaging System (LI-COR, Inc., USA) and quantified with image J.

### Statistical analysis

Data are expressed as mean ± SD, *n* = 3. One-way ANOVA or student *t*-tests were used for statistical analysis. To avoid bias, all statistical analyses were performed blind. The significance level is established in *P < 0.05, **P < 0.01, and ***P < 0.001.

## Supplementary information

Supplementary Figures

Original Data File

## Data Availability

The corresponding author will provide the original data used to support the findings of this study upon reasonable request.
