# Correction: Periplogenin attenuates LPS-mediated inflammatory osteolysis through the suppression of osteoclastogenesis via reducing the NF-κB and MAPK signaling pathways

**DOI:** 10.1038/s41420-026-03007-z

**Published:** 2026-03-30

**Authors:** Kai Gan, Haoyu Lian, Tao Yang, Jian Huang, Junchun Chen, Yuangang Su, Jinmin Zhao, Jiake Xu, Qian Liu

**Affiliations:** 1 Guangxi Key Laboratory of Regenerative Medicine, Orthopaedic Department, The First Affiliated Hospital of Guangxi Medical University, Nanning, Guangxi 530021 China; 2 Collaborative Innovation Centre of Regenerative Medicine and Medical BioResource Development and Application Co-constructed by the Province and Ministry, Life Sciences Institute, Guangxi Medical University, Nanning, Guangxi 530021 China; 3 Faculty of Pharmaceutical Sciences, Shenzhen Institute of Advanced Technology, Chinese Academy of Sciences, Shenzhen, 518000 China

Correction to: *Cell Death Discovery* 10.1038/s41420-024-01856-0, published online 17 February 2024

In this article, we incidentally mis-placed pictures from a different file due to a careless overlook during our figure ppt slide preparation.

Incorrect figure
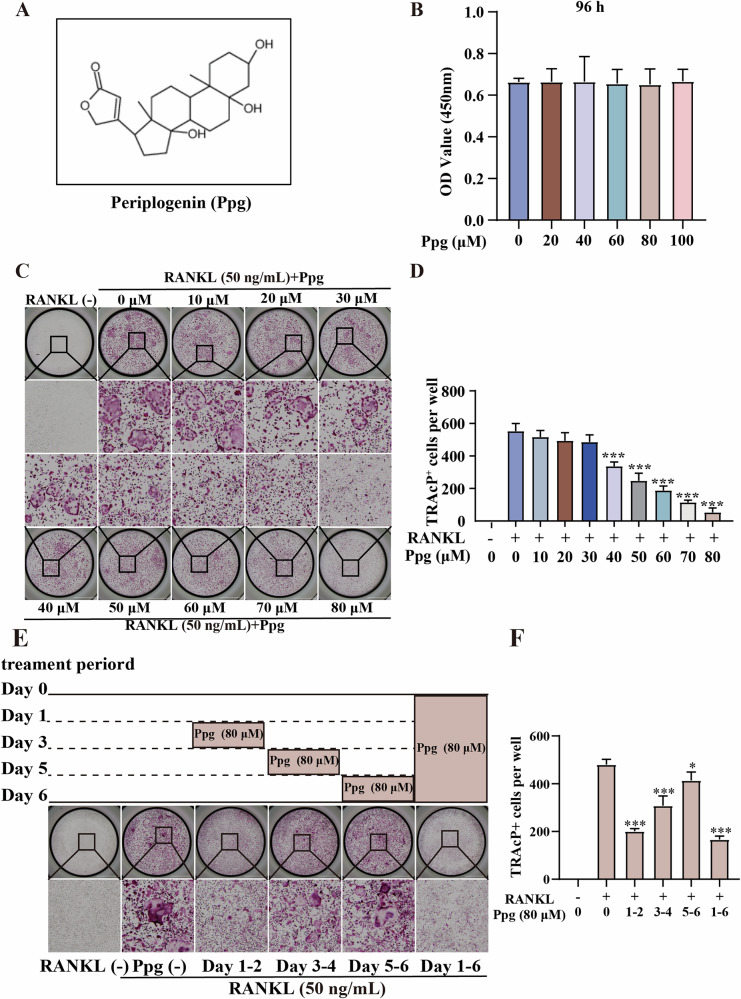


Correct figure
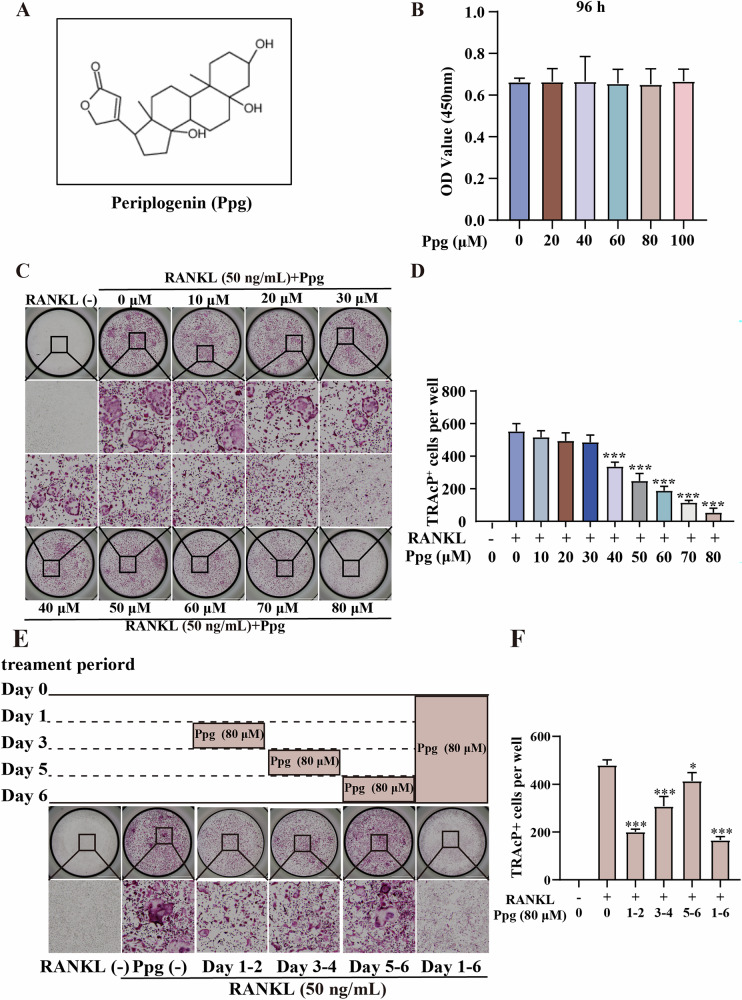


Incorrect figure
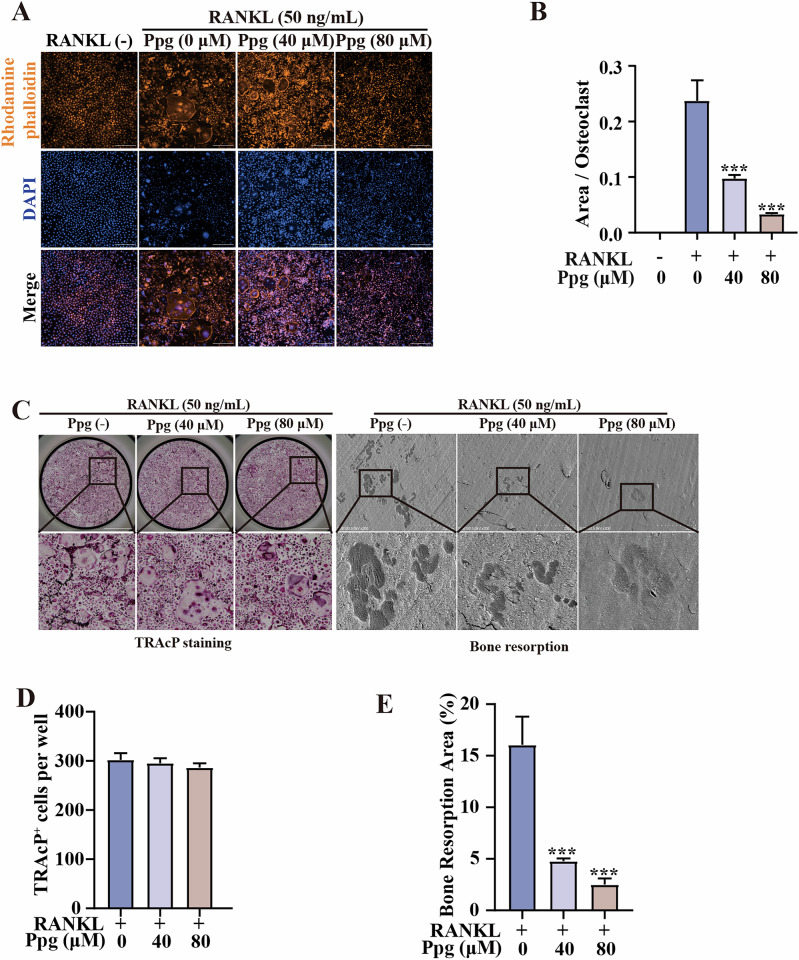


Correct figure
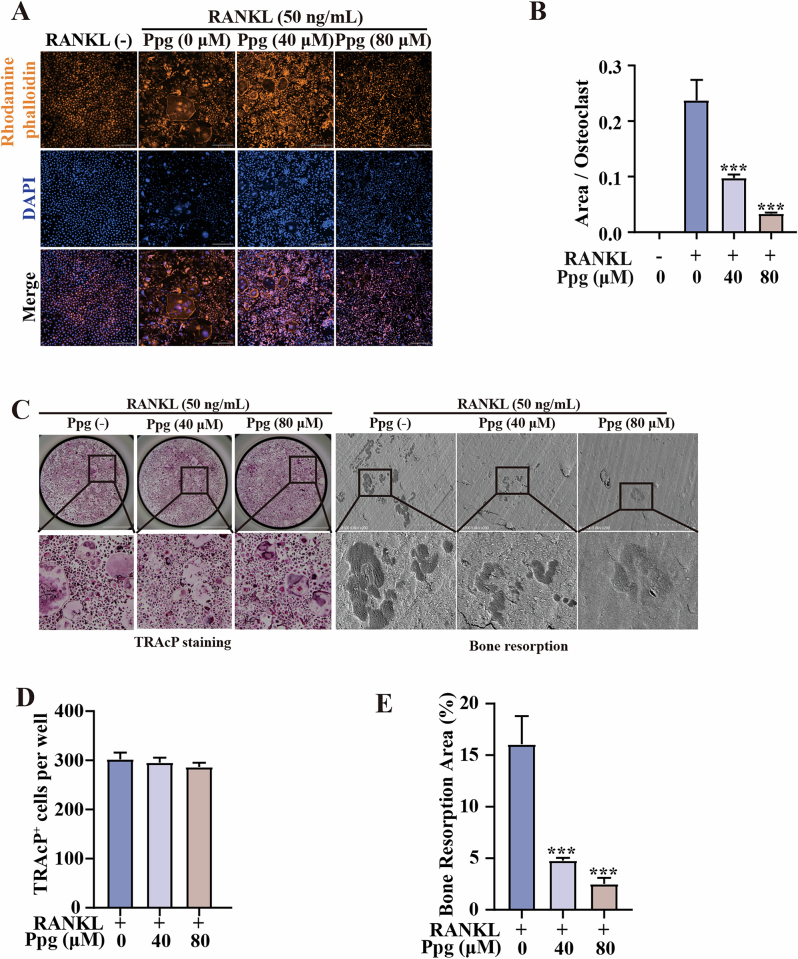


The original article has been corrected.

