# Supplementary material for: Periplogenin attenuates LPS-mediated inflammatory osteolysis through the suppression of osteoclastogenesis via reducing the NF-κB and MAPK signaling pathways
Source: Cell Death Discov. 2024 Feb 17;10:86. doi: 10.1038/s41420-024-01856-0 (PMC10874423; doi:10.1038/s41420-024-01856-0)
Supplement: Supplementary file 1 — Supplementary Figures [file 41420_2024_1856_MOESM1_ESM.docx]

**Electronic Supplementary Figures**

**Supplementary Figures S1 – S3.**


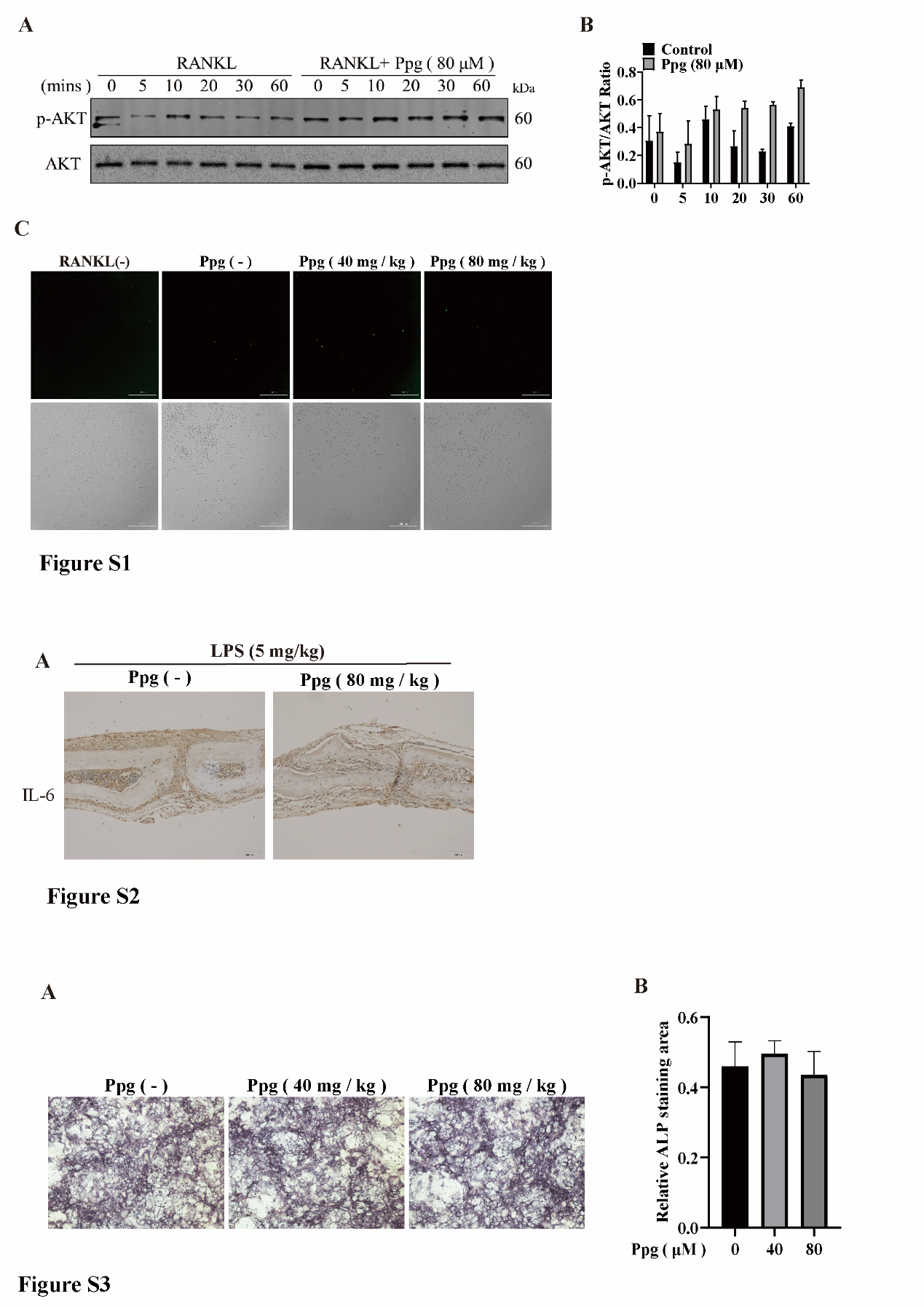


**Figure S1.** **Ppg does not affect the pro-inflammatory cytokine il-6 *in vivo*.** (A) Representative images of il-6 immunohistochemical staining *in vivo.*


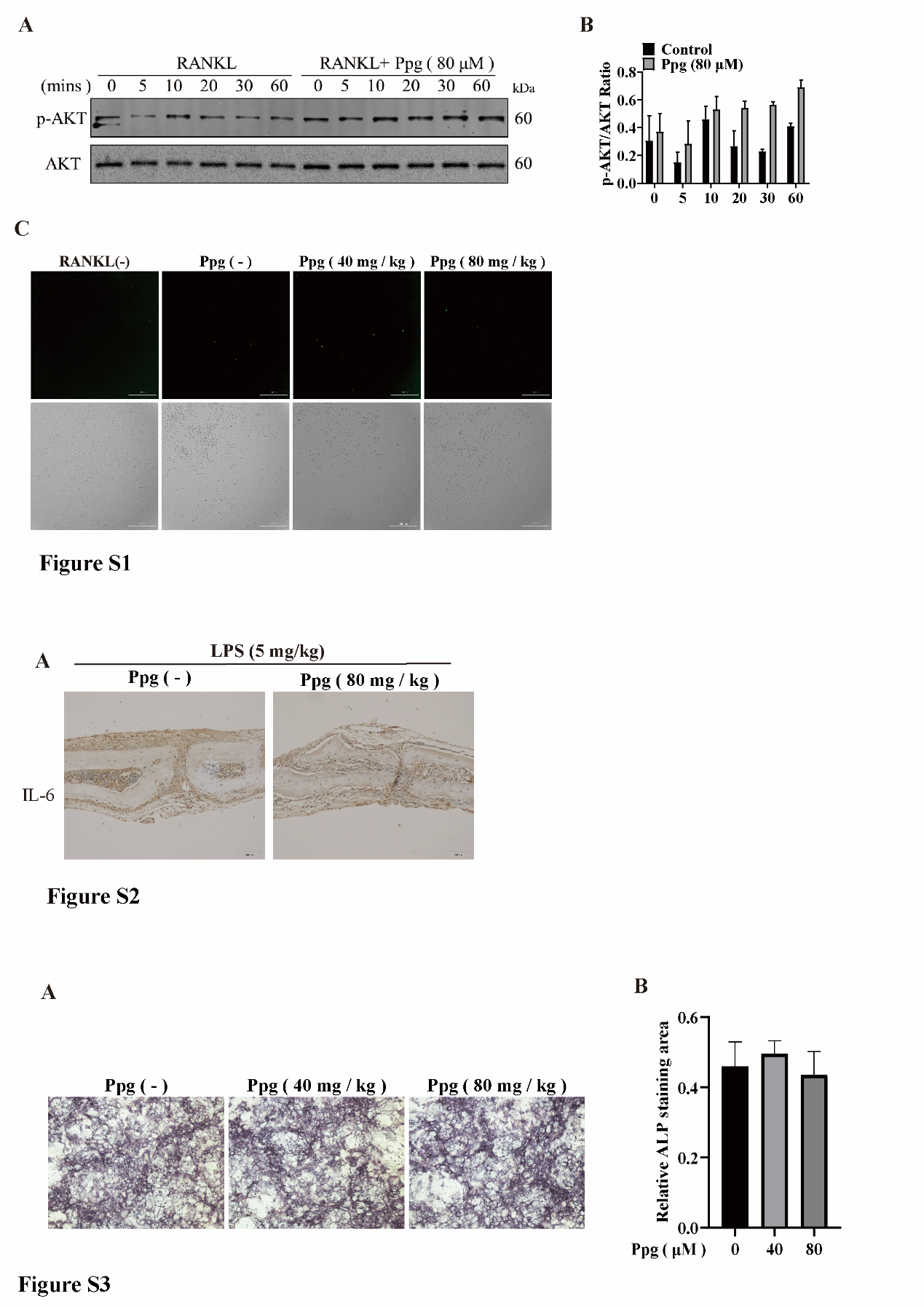
**Figure S2. Ppg** **does not suppress PI3K-AKT and ROS signaling pathways during osteoclastogenesis.** (A) The expression levels of p-AKT and AKT were measured by western-blot. (B) The ratio of intensity of the above proteins expression was indicated. (C) BMMs were treated with or without Ppg for 48 h, and then intracellular ROS levels were measured by DCFH-DA assay. * p < 0.05, ** p < 0.01, *** p < 0.001. All data are expressed as mean ± SD.


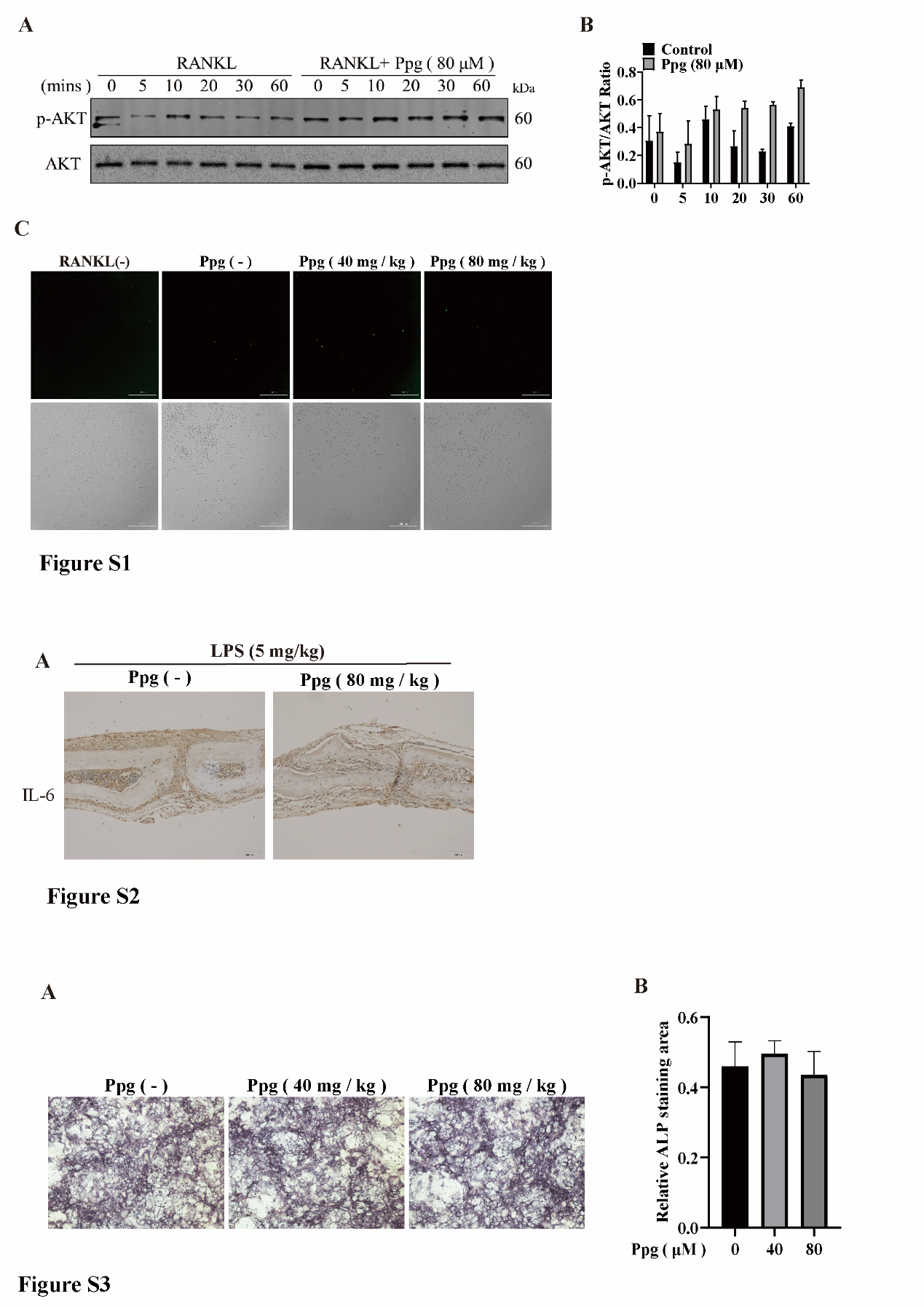
**Figure S3.** **Ppg does not affect bone formation.** (A) Representative images of ALP staining in the presence of Ppg (40 μM and 80 μM) for 7 days. (B) Quantification intensity of the ALP staining after Ppg treatment. * p < 0.05, ** p < 0.01, *** p < 0.001. All data are expressed as mean ± SD.
