# Supplementary figures and images for: Periplogenin attenuates LPS-mediated inflammatory osteolysis through the suppression of osteoclastogenesis via reducing the NF-κB and MAPK signaling pathways
Source: Cell Death Discov. 2024 Feb 17;10:86. doi: 10.1038/s41420-024-01856-0 (PMC10874423; doi:10.1038/s41420-024-01856-0)

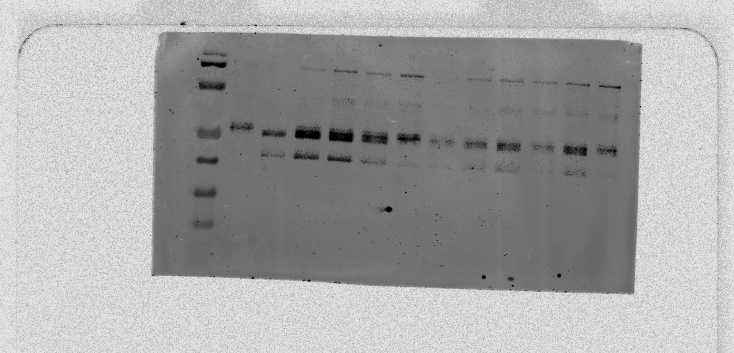

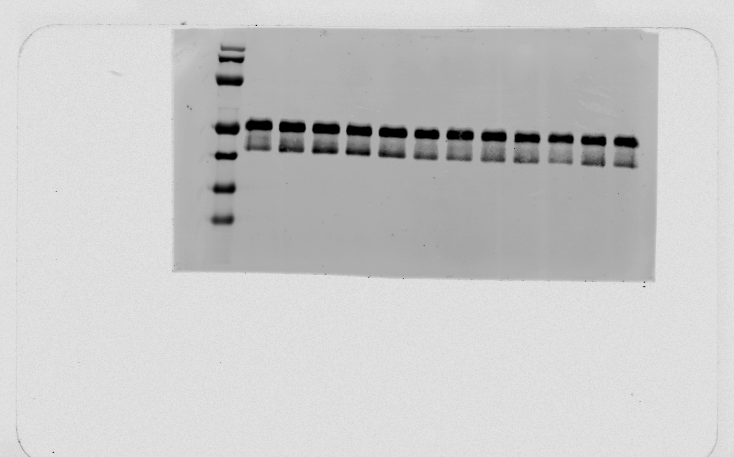

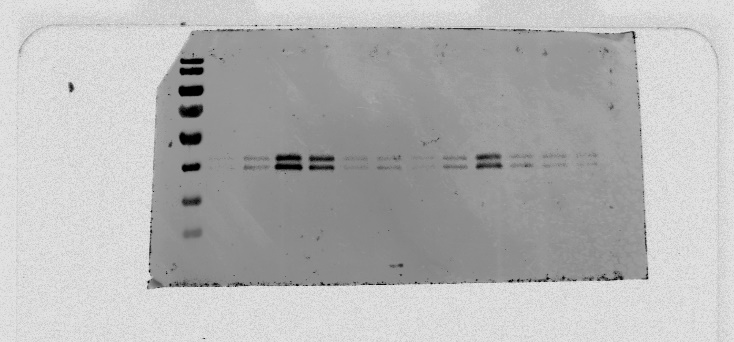

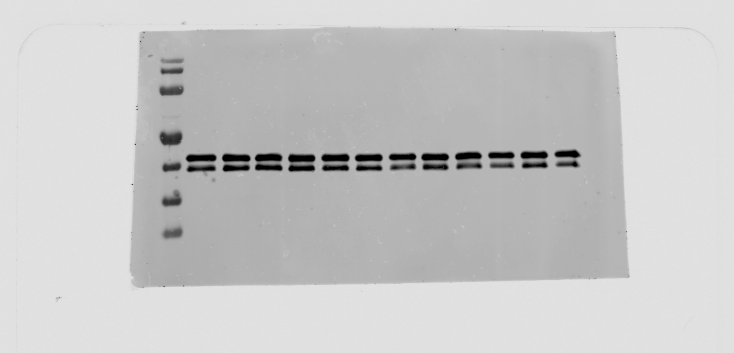
p-ERK ERK

p-JNK JNK

p-P38 P38


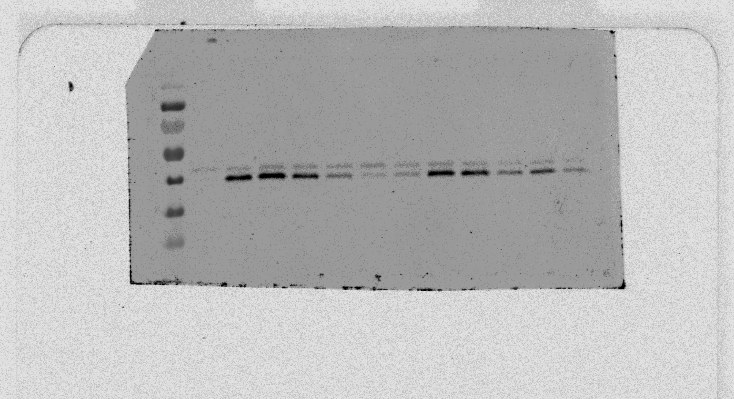

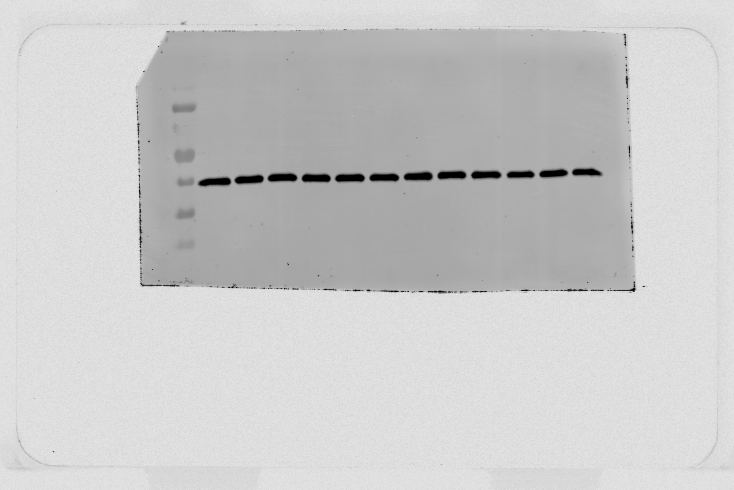


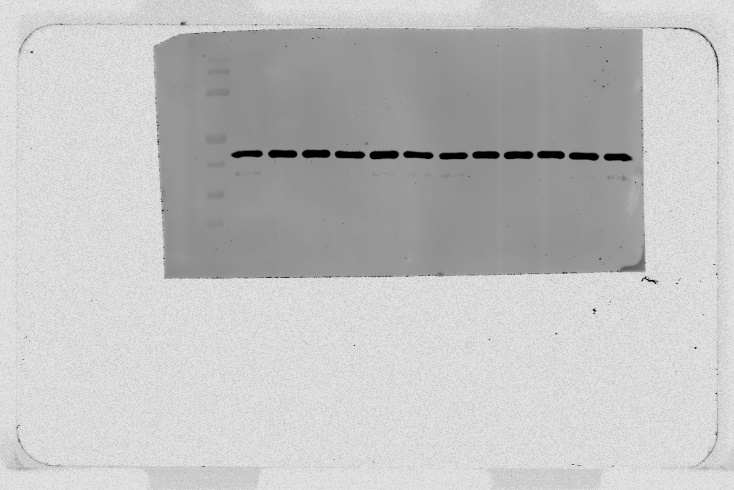

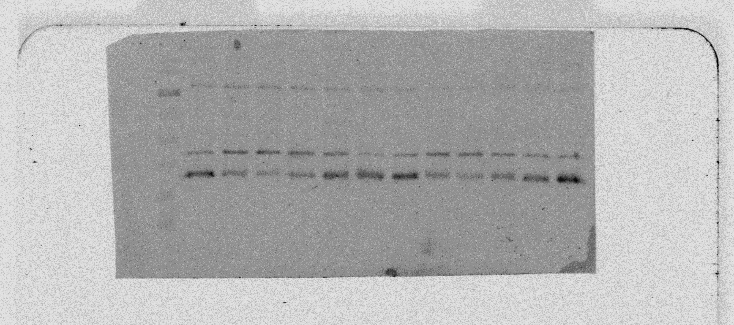
 IκBα β-actin

p-P65 P65


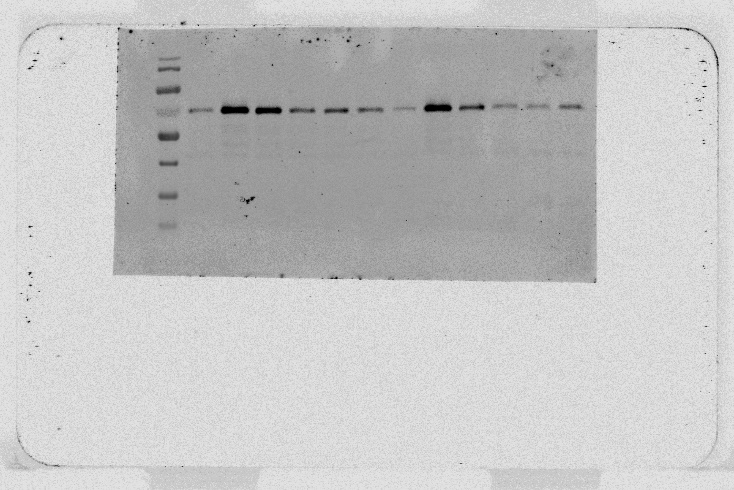


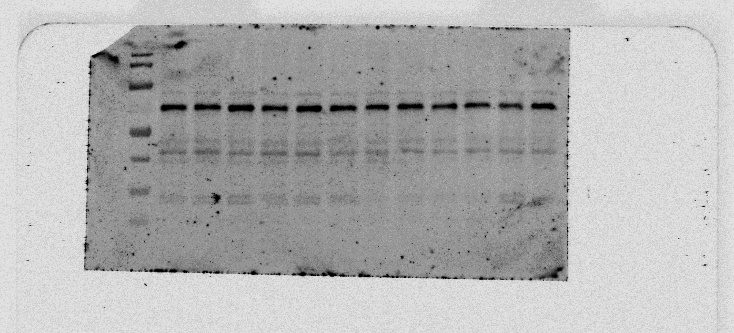


p-AKT AKT


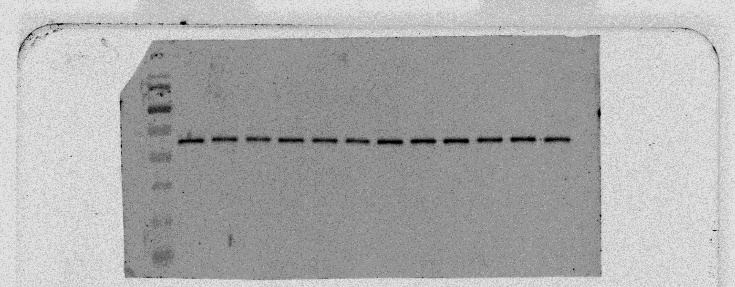

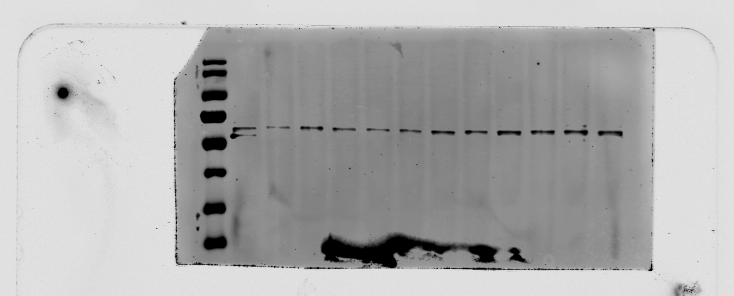


NFATc1


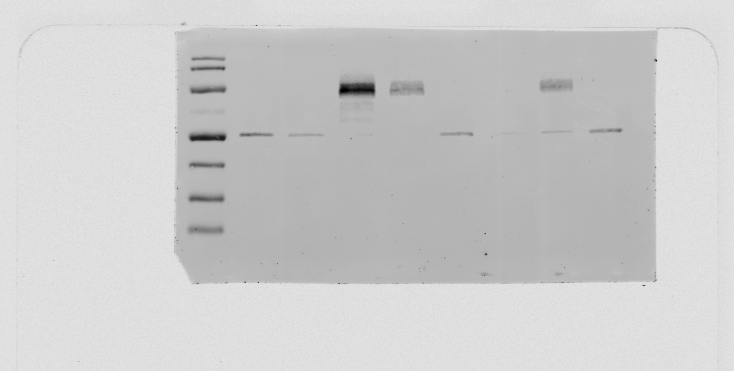


c-Fos


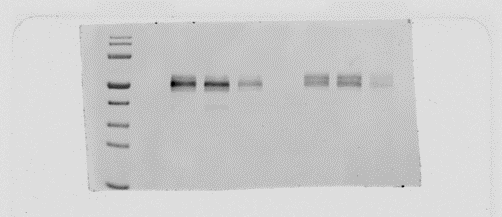


CTSK


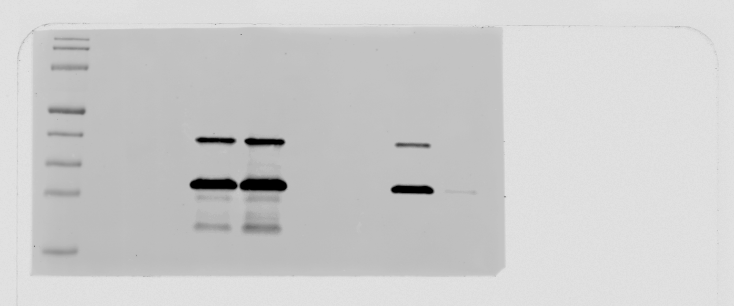


Atp6v0d2


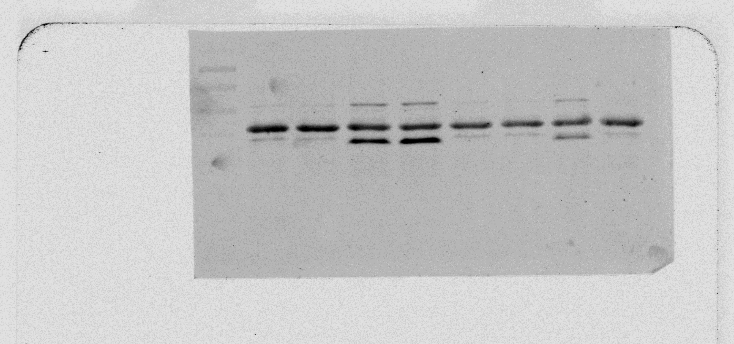


β-actin


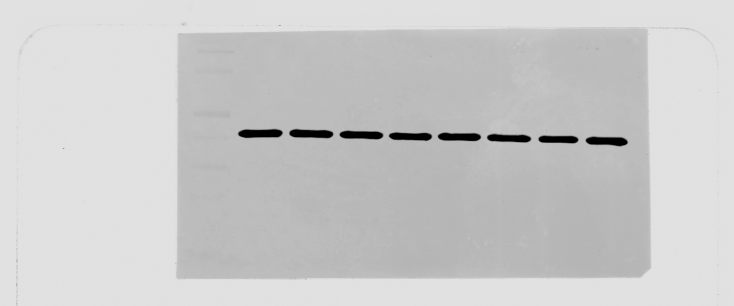

Supplement: Supplementary file 2 — Original Data File [file 41420_2024_1856_MOESM2_ESM.docx]
